# Clinicoprognostic implications of increased serum levels of vascular endothelial growth factor and basic fibroblastic growth factor in early B-cell chronic lymphocytic leukaemia

**DOI:** 10.1038/sj.bjc.6600022

**Published:** 2002-01-07

**Authors:** S Molica, G Vitelli, D Levato, A Ricciotti, G Digiesi

**Affiliations:** Department of Hematology, Azienda Ospedaliera ‘Pugliese-Ciaccio’, Catanzaro, Italy; Department of Oncology, Azienda Ospedaliera ‘Pugliese-Ciaccio’, Catanzaro, Italy; Clinical Pathology Service, Istituto ‘Regina Elena’ IRCCS, Roma, Italy

**Keywords:** vascular endothelial growth factor, basic fibroblast growth factor, Stage A chronic lymphocytic leukaemia, disease- progression

## Abstract

To assess the relative merit of increased serum levels of vascular endothelial growth factor and basic fibroblastic growth factor in predicting the risk of disease progression of patients with early B-cell chronic lymphocytic leukaemia we analyzed 81 Binet stage A patients whose sera were taken at the time of diagnosis and evaluated for the presence of vascular endothelial growth factor and basic fibroblast growth factor using an enzyme-linked immunosorbent assay. Serum levels of vascular endothelial growth factor positively correlated with Rai sub-stages (*P*=0.03), peripheral blood lymphocytosis (*P*=0.03), bone marrow histology (*P*=0.04) and β2-microglobulin (β2-m) (*P*=0.006). When dealing with basic fibroblast growth factor only a correlation with Rai sub-stages (*P*=0.02) could be found. Different cut-offs set on the basis of a stratification in quartiles, failed to demonstrate any correlation between serum levels of basic fibroblast growth factor and disease progression. In contrast, patients with increased serum levels of vascular endothelial growth factor (above median value, 203 pg ml^−1^) had a three times increased risk of disease progression, although, in multivariate analysis only Rai sub-stages (*P*=0.0001) and lymphocyte doubling time (*P*=0.002) retained their prognostic significance. Low levels of vascular endothelial growth factor were indicative of good clinical outcome in the subgroup of patients with either low (*P*=0.02) or high (*P*=0.03) β2-m concentration. Finally, the highest prognostic power was obtained when serum vascular endothelial growth factor and β2-m were examined in combination. Median of progression-free survival of patients who had both serum vascular endothelial growth factor and β2-m higher than median value was only 13 months, in contrast median progression-free survival of patients with one marker increased (i.e. above the 50th percentile) was 40 months. Patients with both markers below the median experienced the best clinical outcome (median progression-free survival not reached at 40 months). In conclusion, serum levels of either vascular endothelial growth factor or basic fibroblast growth factor are high in patients with early chronic lymphocytic leukaemia, however, only vascular endothelial growth factor predicts behaviour of disease and helps to refine the prognosis of stage A patients.

*British Journal of Cancer* (2002) **86**, 31–35. DOI: 10.1038/sj/bjc/6600022 www.bjcancer.com

© 2002 The Cancer Research Campaign

## 

In patients with cancer, high serum concentrations of VEGF are associated with several unfavourable clinical parameters such as short tumour volume doubling time, progressive disease, extensive disease and poor survival (Dirix *et al*, 1996, 1997; Salven *et al*, 1997a,b Kitamura *et al*, 1998; Tempfer *et al*, 1998; Ugurel *et al*, 2001). Similarly, high concentrations of b-FGF have been detected in either urine or serum of cancer patients and associated with tumour bulk in head and neck or with a short tumour volume doubling time in colorectal cancer (O'Brien *et al*, 1995; Leunig *et al*, 1998; Kumar *et al*, 1998). More recently, high pretreatment level of b-FGF has been found an independent predictor of poor prognosis in non-Hodgkin lymphomas (NHLs) (Salven *et al*, 1999b). Furthermore, the simultaneous measurements of VEGF and b-FGF provide prognostic information additional to international prognostic index (IPI) (Salven *et al*, 2000).

Recent studies indicate that angiogenesis may also be involved in the pathogenesis of B-cell chronic lymphocytic leukaemia (CLL) (Aguayo *et al*, 2000; Kini *et al*, 2000). Elevated pretreatment levels of b-FGF correlate with a more advanced clinical stage and with resistance to chemotherapy (Menzel *et al*, 1996; Konig *et al*, 1997), while low cellular and high serum levels of VEGF are associated with a poor clinical outcome (Molica *et al*, 1999; Aguayo *et al*, 2000). Although these data lend support to the idea that angiogenic cytokines may play a role in the leukaemogenic process of CLL, the relative merit of VEGF and b-FGF in predicting the outcome of disease has not been assessed thus far.

In this study we expanded on previous observation by evaluating VEGF and b-FGF in the serum of patients with early CLL (i.e., Binet stage A) (Binet *et al*, 1981). In this subset of patients angiogenic cytokines were investigated as indicator of disease-progression (DP), an event which has an important impact on overall survival of CLL patients.

## PATIENTS AND METHODS

### CLL patients

Eighty-one CLL Binet stage A (Binet *et al*, 1981) patients treated at our institution form the basis of this study. Their median age was 66 years (range, 44–82 years) and the male to female ratio 49 to 32 years. Routine laboratory studies of these patients were performed at diagnosis and consisted of complete blood count with differential, platelet count, and blood chemistry including β-2 microglobulin (β-2m) and LDH, immunophenotyping to establish the diagnosis of typical CLL (Cheson *et al*, 1996). Physical examination, chest X-ray, and abdominal ultrasound were performed in all instances.

Patients were also staged according to the Rai (Rai *et al*, 1975) staging system and distributed as follows: stage 0, 54 patients (66.6%); stage I, seven patients (8.6%); stage II, 20 patients (24.6%). BM biopsies were performed at the time of diagnosis in 63 (77.7%) out of 81 patients and the pattern of BM involvement was evaluated according to the criteria suggested by Rozman *et al* (1984). Lymphocyte doubling time (LDT), available in 73 patients (90.1%) patients followed-up for a period >12 months was assessed according to the method of Montserrat *et al* (1986).

#### Determination of serum levels of VEGF and b-FGF

Peripheral venous blood samples were collected in sterile tubes at the time of CLL diagnosis, centrifugated at 2000 **g** for 10 min and stored at −70°C. VEGF and b-FGF concentrations were determinated by using a quantitative ELISA technique (Human VEGF and human b-FGF, Quantikine™ and Quantikine®; R&D Systems, Minneapolis, MN, USA) as previously reported (Molica *et al*, 1999). Concentrations were reported as pg ml^−1^. The sensitivity of test was less than 5 pg ml^−1^ for VEGF and less than 3 pg ml^−1^ for b-FGF. The coefficient of variation (CV) of interassay determinations reported by manufacturer vary from 6.2–8.8% and the intra-assay variation from 2 to 9% for VEGF. As far as b-FGF is concerned, the CV of interassay determinations reported vary from 7.4 and 9.1% while the intra-assay variation from 3 to 9.7%

Serum samples from 63 healthy blood donors (40 males and 23 females) without any evidence of diseases (i.e., liver dysfunction, diabetes, etc.) whose age ranged between 40 and 65 years were analyzed for VEGF presence. Given the consistent difference in the serum levels of b-FGF between CLL patients and healthy donors, for such a comparison only 20 normal controls were considered sufficient. The previously correlation between serum VEGF levels and platelet count (Banks *et al*, 1998; Fuhrmann-Benzakein *et al*, 2000) prompted us to measure VEGF concentration on either plasma or serum in 30 unselected CLL Binet stage A patients. Different modalities of sample collection did not prevent us from finding a close correlation between circulating levels of VRGF and platelet count on either serum (*r*=0.524; *P*<0.0001) or plasma (*r*=0.449; *P*<0.0001).

#### Other assays

β-2 m and LDH serum levels were determinated as previously described (Molica *et al*, 1999).

### Clinical studies and disease-progression evaluation

Serum levels of either VEGF or b-FGF were correlated with main clinical variables such as Rai clinical sub-stages, BM histology, absolute PB lymphocytosis, LDT, LDH and β2-m. Furthermore, VEGF and b-FGF were investigated as predictor of clinical outcome. To this purpose we used as end-point disease-progression (DP) defined as the appearance of the upper stages according to the Binet (Binet *et al*, 1981) classification during the treatment-free period. As previously reported, DP has an important impact on overall survival of CLL patients in early disease stage (Molica, 1991). Therefore, DP can surrogate overall survival, thus shortening the duration of clinical studies in CLL.

### Statistical methods

Pearson correlation, Spearman correlation and the corrected χ^2^ test were applied to compare groups. Progression-free survival (PFS) curves were plotted according to the method of Kaplan–Meier and compared with the log-rank test. To evaluate the relative significance of some prognostic factors, the multiple regression model of Cox was applied . The set variables analyzed were as follows: stage according to Rai staging (0 *vs* I–II) , LDT (<12 *vs* >12 months), absolute lymphocyte count (<20 *vs* >20×10^9^/l), LDH (<425 *vs* 425 U l^−1^), β-2 m (<2.89 *vs* >2.89 mg l^−1^), sVEGF (<203 *vs* >203 ng ml^−1^). The cut-off values of four continuous variables was determinated on the basis of median value of entire patient population.

## RESULTS

### Serum VEGF and b-FGF concentrations in stage A B-cell CLL patients

**Figure 1:**
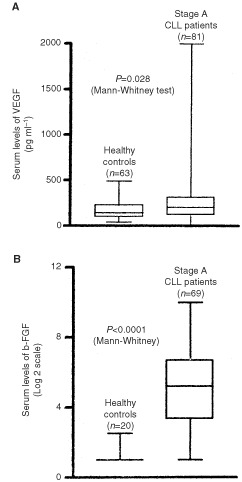
Serum levels of VEGF and b-FGF. This box plot compares median levels of VEGF (**A**) and b-FGF (**B**) in stage A B-cell CLL and healthy controls.

As shown in Figure 1

, serum levels of VEGF and b-FGF were higher in stage A CLL patients in comparison to healthy controls (*P*=0.028 and *P*<0.0001, respectively).

The relationship between VEGF and b-FGF serum levels and main clinico-hematological features of CLL is presented in Table 1

**Table 1:**
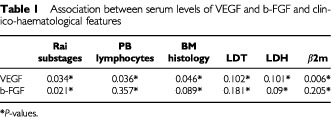
Association between serum levels of VEGF and b-FGF and clinico-haematological features

. Serum levels of VEGF positively correlated with Rai substages (*P*=0.03), peripheral blood lymphocytosis (*P*=0.03), BM histology (*P*=0.04), β2-m (*P*=0.006). In contrast, only a correlation with Rai clinical substages (*P*=0.02) could be found when dealing with b-FGF.

### Serum levels of VEGF and b-FGF and the risk of disease-progression

**Figure 2:**
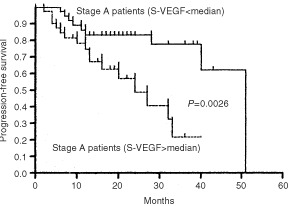
Progression-free survival (PFS) curves of stage A patients stratified on the basis of median value of sVEGF (i.e., 203 pg ml^−1^).

The impact of VEGF and b-FGF on the clinical outcome of disease was evaluated using as end-point DP. After a median follow-up time of 15 months (range, 2–51 months), 26 out of 81 (32%) stage A patients moved to a more advanced clinical stage (13 from stage A to B, 13 from stage A to C). Different cut-offs set on the basis of a stratification in quartiles failed to demonstrate any correlation between serum levels of b-FGF and DP (data not shown). In contrast, when dealing with VEGF clear-cut differences could be appreciated at a median value (i.e., 203 pg ml^−1^). Median time of PFS was 24 months for patients with VEGF serum levels above the median value, whereas it was 51 months for patients with concentration of VEGF below median value (*P*=0.002; hazard risk (HR), 0.321; 95% CI, 0.124–0.643) (Figure 2

). The correlation between VEGF serum levels and platelet count prompted us to evaluate whether platelet count had any prognostic value in the current series. No association was found between platelet count and the risk of DP (*P*=0.09), thus it appears that circulating levels of VEGF are high in stage A CLL patients with poor outcome and such a finding is an independent prognostic factor irrespective of platelet count. In terms of relative risk (RR), patients with higher levels of VEGF had a three times increased risk of DP (RR, 3.27; 95% CI, 1.23–8.58) which was lower than relative risks associated with Rai substages (RR, 7.39; 95% CI, 2.66–20.49), LDT (RR, 7.20; 95% CI, 2.72–18.92) and BM histology (RR, 8.71; 95% CI, 2.29–32.7). As a matter of fact, in the multivariate analysis only Rai substages (*P*=0.0001) and LDT (*P*=0.002) retained their prognostic significance (Table 2

**Table 2:**
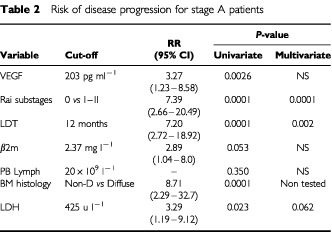
Risk of disease progression for stage A patients

).

**Figure 3:**
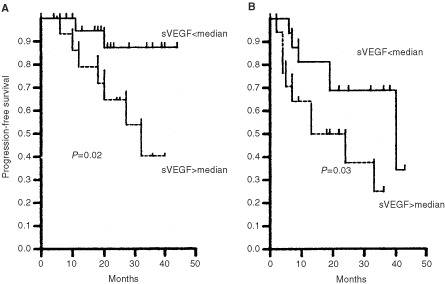
Progression-free survival (PFS) of patients with low (**A**) (i.e., less than 2.73 mg l^−1^) or high (**B**) β2-m concentration stratified on the basis of median value of serum VEGF concentration.

The effect of serum VEGF levels on DP was evaluated after dividing patients into groups with high β2-m (poor prognostic subgroup) and low β2-m (good prognostic subgroup) concentrations. For such a stratification we used as cut-off the median β2-m value (i.e., 2.73 mg l^−1^). Serum levels of VEGF higher than median value (i.e., 203 pg ml^−1^) identified patients with worse outcome in the subgroup with either low (HR, 0.200; 95% CI, 0.057–0.826; *P*=0.02; Figure 3A

) or high β2-m (HR, 0.377; 95% CI, 0.114–0.947; *P*=0.03; Figure 3B) concentration.

**Figure 4:**
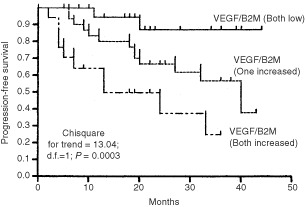
Progression-free survival (PFS) of 81 stage A patients by serum concentration of β2-m and VEGF. The cut-offs used were median serum levels for both markers (VEGF, 203 pg ml^−1^; β2-m, 2.72 mg l^−1^). The plotted PFS lines from the top represent: Patients with both markers low; patients with one marker increased; patients with both markers increased.

Finally, given the partial independence of VEGF from β2-m the combined effect of these markers on DP of stage A patients was studied. Patients with both markers increased (above the median values) had the worst clinical outcome (median of PFS, 13 months) while those with both markers low the best one (median of PFS not reached at 44 months); in between there was an intermediate- risk group characterized for having only one marker increased (median of PFS, 40 months; χ^2^ for trend=13.04, d.f.=1, *P*=0.0003; Figure 4

).

## DISCUSSION

Results of the current study show that serum concentrations of either VEGF or b-FGF are increased in the serum of stage A CLL patients but only serum levels of VEGF reflect clinico-haematological features of tumour mass such as advanced Rai clinical substages, high PB lymphocytosis, increased β2-m and diffuse BM histology.

Most circulating VEGF is found in platelets and leukocytes and released from the blood cells during the coagulation process (Banks *et al*, 1998). However, also after taking into account leukocyte and platelet counts circulating levels of VEGF are higher in cancer patients than in healthy persons (Salven *et al*, 1999a). The findings of present study are in keeping with this because platelet count did not have any impact on the risk of DP, whereas the serum VEGF concentration was associated with an increased risk to progress to a more advanced clinical stage. Those patients whose concentrations of VEGF were higher than median value had a three times increased relative risk (RR) of DP. This RR was lower than the relative risks associated with other well-defined prognostic variable in CLL such as Rai substages and LDT as a consequence serum levels of VEGF were not significant in the multivariate analysis. Nonetheless, VEGF serum levels may be useful to refine prognosis of patients with early CLL; higher levels of sVEGF were associated with a shorter time of DP in Binet stage A patients with either low or high β2-m levels.

The partial independence of sVEGF from β2-m prompted us to use these markers as a combined parameter and such a combination clearly determinated three prognostic groups. So different serological markers that contribute individually to prognosis of CLL such as β2-m and VEGF, integrating different clinical and biological aspects of CLL, provide prognostic information superior to those of a single marker.

To identify patients at higher risk of DP a number of independent prognostic factors such as LDT, soluble CD23, β2-m and serum thymidine kinase have been proposed in patients with early CLL (Molica, 2001). More recently, genotypic aberrations have been reported as important independent predictors of DP and survival and two independent studies indicate that IgH gene mutation status has relevant prognostic implications (Hamblin *et al*, 1999; Damle *et al*, 1999; Dohner *et al*, 2000). As a surrogate of IgH gene evaluation Damle *et al* (1999) have suggested to take into account CD38 expression which stands as an excellent prognostic marker in CLL (Ibrahim *et al*, 2001). However, it is not clear what is the applicability of all these markers in the setting of clinical practice. When dealing with serum markers such as VEGF, a major problem is the lack of standardized methods for their measurements. As a result, prognostic cut-off may vary thus avoiding the general applicability of many serum markers in the day-by-day practice. For these reasons, the evaluation of serum levels of VEGF should not be considered standard practice in patients with CLL, rather, as shown in the present paper, VEGF might improve the assessment of individual patient prognosis in early CLL. Finally, given the use of DP as a survival surrogate end-point and the relatively short follow-up our data should be taken with caution. Furthermore, longitudinal studies of VEGF determinations are needed in order to understand whether the progression of early CLL takes place in two phases, prevascular and vascular, respectively.
